# Development of a graphical user interface for automatic separation of human voice from Doppler ultrasound audio in diving experiments

**DOI:** 10.1371/journal.pone.0283953

**Published:** 2023-08-10

**Authors:** Arian Azarang, S. Lesley Blogg, Joshua Currens, Rachel M. Lance, Richard E. Moon, Peter Lindholm, Virginie Papadopoulou

**Affiliations:** 1 Biomedical Engineering Department of University of North Carolina at Chapel Hill, Chapel Hill, NC, United States of America; 2 SLB Consulting, Winton, Cumbria, United Kingdom; 3 Department of Emergency Medicine, School of Medicine, University of California, La Jolla, CA, United States of America; 4 Joint Department of Biomedical Engineering, University of North Carolina at Chapel Hill, Chapel Hill, NC, United States of America; 5 North Carolina State University, Raleigh, NC, United States of America; 6 Center for Hyperbaric Medicine and Environmental Physiology, Duke University, Durham, NC, United States of America; Vinnytsia National Technical University, UKRAINE

## Abstract

Doppler ultrasound (DU) is used in decompression research to detect venous gas emboli in the precordium or subclavian vein, as a marker of decompression stress. This is of relevance to scuba divers, compressed air workers and astronauts to prevent decompression sickness (DCS) that can be caused by these bubbles upon or after a sudden reduction in ambient pressure. Doppler ultrasound data is graded by expert raters on the Kisman-Masurel or Spencer scales that are associated to DCS risk. Meta-analyses, as well as efforts to computer-automate DU grading, both necessitate access to large databases of well-curated and graded data. Leveraging previously collected data is especially important due to the difficulty of repeating large-scale extreme military pressure exposures that were conducted in the 70-90s in austere environments. Historically, DU data (Non-speech) were often captured on cassettes in one-channel audio with superimposed human speech describing the experiment (Speech). Digitizing and separating these audio files is currently a lengthy, manual task. In this paper, we develop a graphical user interface (GUI) to perform automatic speech recognition and aid in Non-speech and Speech separation. This constitutes the first study incorporating speech processing technology in the field of diving research. If successful, it has the potential to significantly accelerate the reuse of previously-acquired datasets. The recognition task incorporates the Google speech recognizer to detect the presence of human voice activity together with corresponding timestamps. The detected human speech is then separated from the audio Doppler ultrasound within the developed GUI. Several experiments were conducted on recently digitized audio Doppler recordings to corroborate the effectiveness of the developed GUI in recognition and separations tasks, and these are compared to manual labels for Speech timestamps. The following metrics are used to evaluate performance: the average absolute differences between the reference and detected Speech starting points, as well as the percentage of detected Speech over the total duration of the reference Speech. Results have shown the efficacy of the developed GUI in Speech/Non-speech component separation.

## Introduction

Automatic speech recognition [1–3] refers to the problem of getting an algorithm to automatically transcribe spoken language and over the last few years [4–6], several automatic speech-to-text (STT) machines have been developed in the literature [7, 8]. When spoken clearly, rudimentary speech recognition software has a limited vocabulary and may only recognize a few words and phrases. More advanced software can deal with natural speech, distinct accents, and several languages [9, 10]. Speech recognition is based on a diverse set of studies in computer science [11], linguistics [12], electrical engineering [13], and medical applications [14]. Many current products and text-focused applications integrate speech recognition functions that make device use easier or hands-free [15–17].

Automated telephone systems and medical dictation software were among the first implementations for speech recognition [18–20]. These are widely used for transcribing, database querying, and commanding computer-based systems, particularly in activities that need specific vocabulary. Personal assistants in automobiles and smartphones, such as Amazon’s Alexa and Apple’s Siri, are also enabled [21, 22].

To ensure that the dialog system provides relevant replies at the proper moment, a highly accurate and rapid speech recognition system must be built. Many cloud-based speech recognition services are available, including the Google Cloud Speech application programming interface (API) [23], IBM Watson Speech to Text [24], and Microsoft Bing Speech API [25]. Google Cloud Speech API, for example, is a Web API that can leverage Google’s speech recognition technology with a high speech recognition rate [26].

The Google speech recognizer uses a neural network to model speech recognition and allows developers to transform audio files into text together with corresponding timestamps in the form of an API that supports 120 different languages around the world. For the best performance of the Google speech recognizer engine, audio files with a 16 kHz sample rate should be used [27]. We have utilized the Google recognition engine to detect the Speech/Non-speech component of audio files. Of greatest importance in the present study is the detection timestamps of human voice activity rather than what has been spoken specifically, as is detailed below.

Doppler ultrasound (DU) is used in decompression research to detect venous gas emboli in the precordium or subclavian vein, as a marker of decompression stress [28, 29]. A 1–3 MHz single element transducer is used to transmit ultrasound and backscattered echoes are recorded either on separate transducer (if the transmit transducer is operating in continuous wave Doppler mode) or on the same transducer (pulsed wave Doppler mode). Venous gas emboli are detected through a frequency shift in the backscattered echoes (reflected from the moving bubbles in blood) and the recorded shifts produce “chirp-like” signal that can be differentiated from other cardiac and blood flow sounds, all in the audible range (i.e. 100 Hz—10 kHz) [28]. This is of relevance to scuba divers, compressed air workers and astronauts to prevent decompression sickness (DCS) that can be caused by these bubbles upon or after a sudden reduction in ambient pressure [30–32]. Doppler ultrasound data is graded by expert raters on the Kisman-Masurel or Spencer scales that are associated to DCS risk.

**Fig 1:**
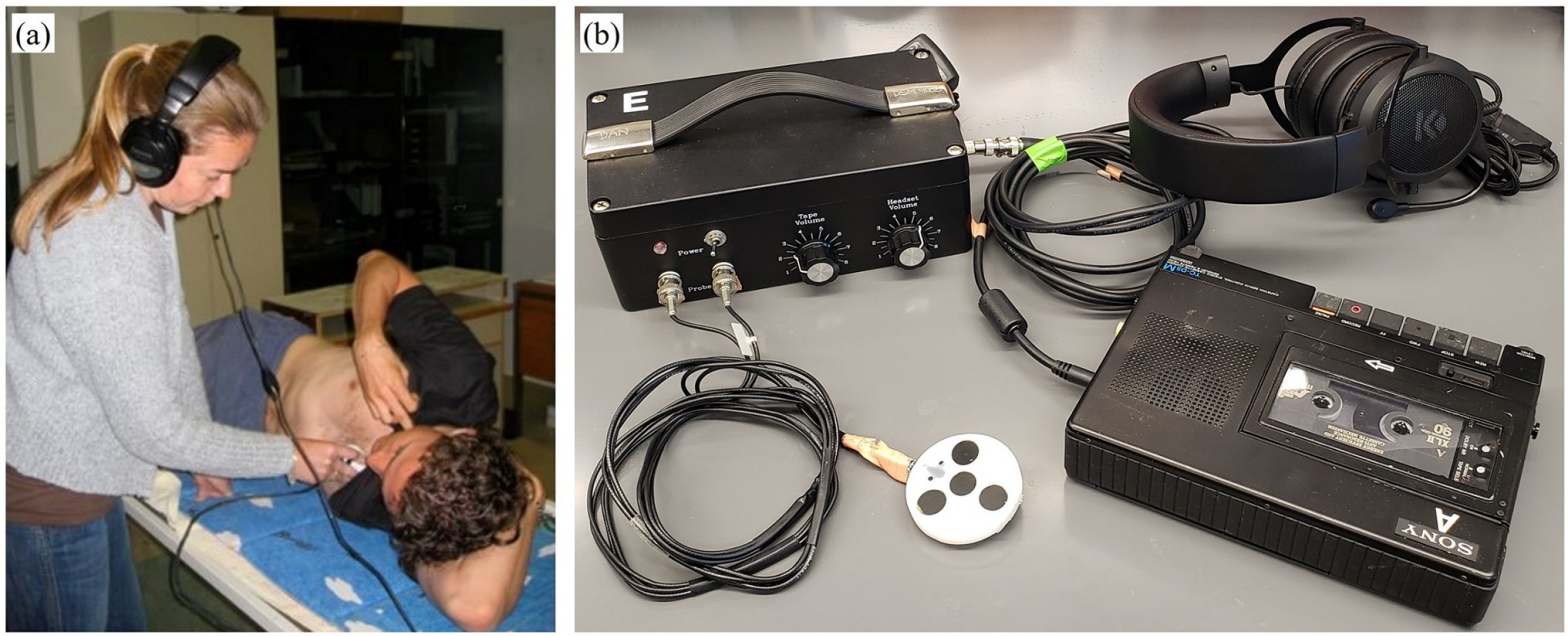
Doppler ultrasound measurement: (a) Doppler technician making a precordial measurement; (b) a portable Doppler ultrasound device (Doppler bubble monitor DBM9008; Techno Scientific Inc., Concord, Canada) with probe and tape recorder for detection. Bubbles are most typically measured over the subclavian vein and the precordial region.

**Fig 2:**
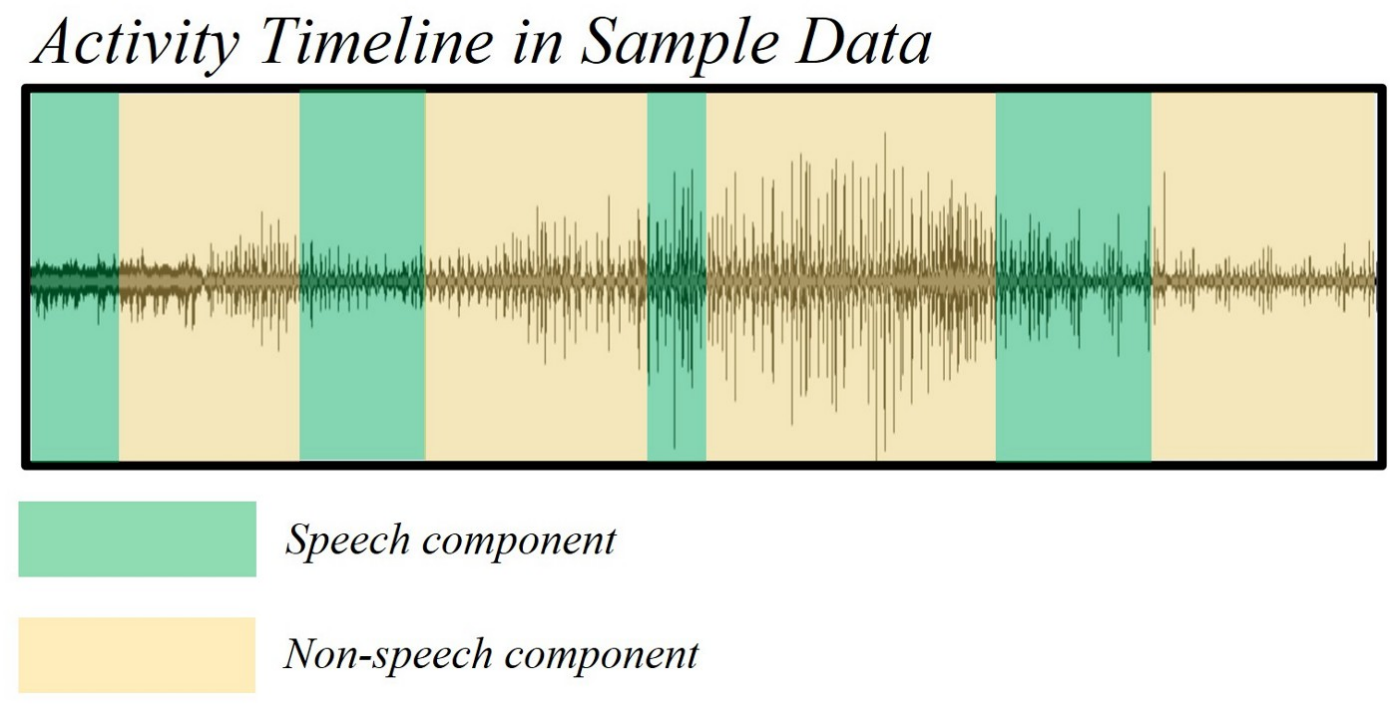
Sample audio signal depicting regions of both Speech and Non-speech components.

Meta-analyses, as well as efforts to computer-automate DU grading, both necessitate access to large databases of well-curated and graded data. A large amount of post-dive audio DU data was recorded in the 1970s and 1980s and recently digitized using audio software tools. In these experiments, DU was recorded on physical tapes, an example of which is shown in Fig 1. This historic data is unique as it was recorded from military divers that accepted a relatively high DCS risk incidence in taking part in such studies [33, 34]. As such, these trials would be near-impossible to repeat nowadays and carry valuable information for modeling and further analysis. However, in numerous recordings, the human voice (Speech component) was recorded together with cardiac signal (Non-speech component) in a single-channel audio. In those cases, tape recordings contain dozens of back-to-back DU data signals and Speech, as depicted in Fig 2. Both Speech (spoken experiment information) and Non-Speech (DU signal) segments are important for data interpretation and DCS modeling. Separation of these component requires advanced signal processing techniques due to the overlaid frequency information in these components. Post-dive DU is graded for VGE presence using several ordinal scales that are associated with DCS probabilities. VGE presence is highly specific but less sensitive, although higher amounts of VGE (above grade 3) are associated with an increase in DCS risk [35–39]. The ability to reconcile those VGE grades to experimental information (subject ID, dive characteristics, DCS outcome, etc) is therefore paramount for database curation and association to DCS outcome.

An example of audio activity timeline for these data is shown in Fig 2. As can be seen from this figure, the human voice activity, as one source of data, is integrated with the audio Doppler, as the second source of data, throughout the entire recording. These data cannot be used for DCS research unless the Speech part and Non-speech are effectively separated. While this task can be performed manually, it is extremely time-consuming and thus impractical for building a large diving research repository of previously acquired data [33].

In this work, we proposed to employ the Google speech recognizer to separate Speech/ Non-Speech components in DU audio files. To conduct a large-scale experiment on recognition and separation tasks, a graphical user interface (GUI) is developed using the license-free Tkinter package of Python. First, the end-user must select the desired audio file to be separated. Then, after recognition of the long human voice component (as a practical assumption, Speech components above three seconds were considered long), the corresponding timestamps of the Speech part were used to apply the separation process. During the recognition process, the end-user was notified by the progress of the program. Once the recognition is finished, the end-user must corroborate the effectiveness of the separation process before saving the output of the GUI. The separated Speech component together with its spectrogram (for visual interpretation) is displayed for the end-user information.

## Materials and methods

In this section, the methodology of the speech recognition and separation is provided together with the GUI structure and functionality of the buttons. Moreover, the dataset used and performance metrics are provided at the end of the section.

### A. Speech recognition and separation methods

**Fig 3:**
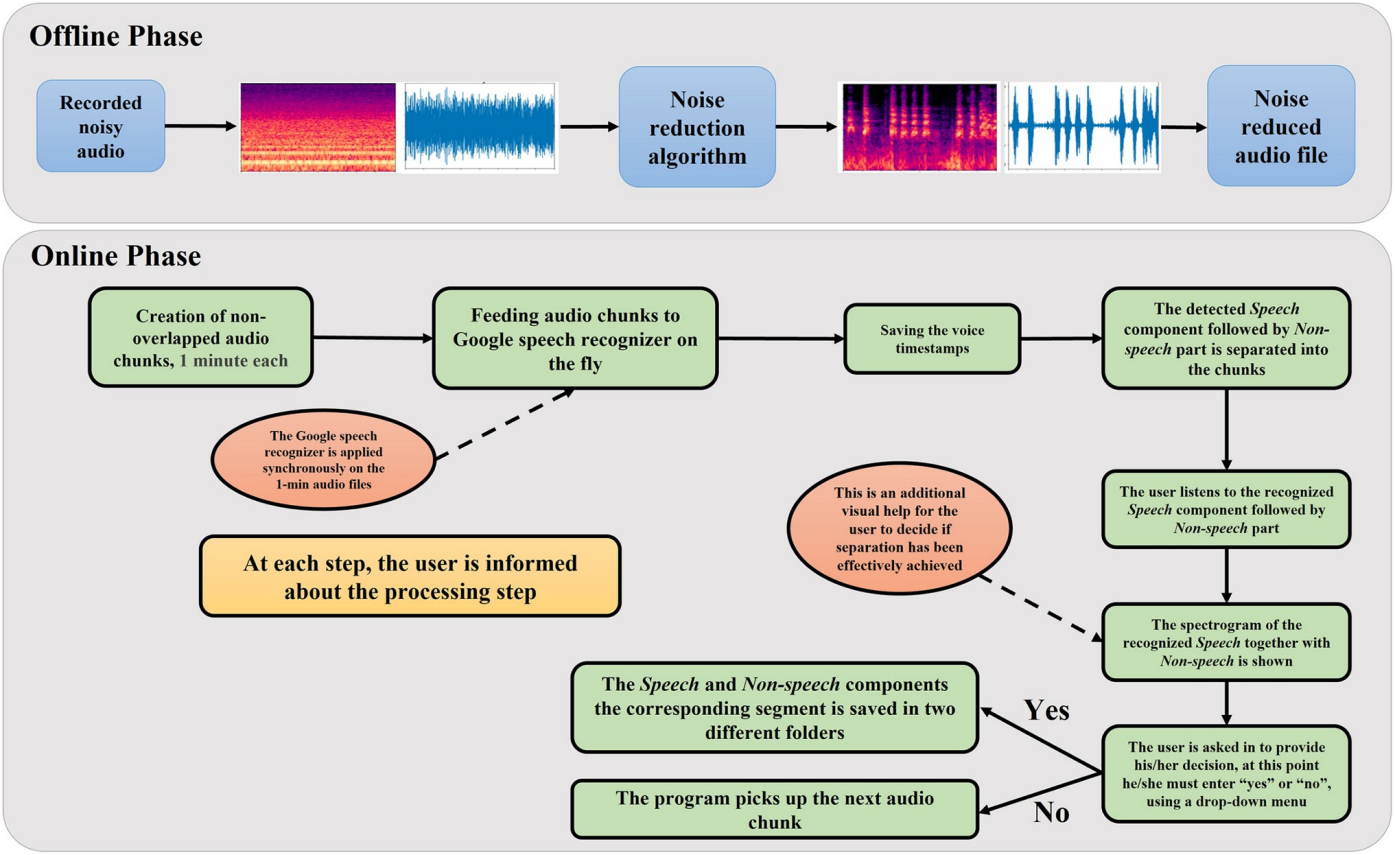
The complete processing steps for speech detection and separation in audio Doppler ultrasound data.

The block diagram of the developed method is depicted in Fig 3. It has been divided into offline and online phases. As a preliminary step during the offline phase, the noise reduction algorithm was applied to the dataset to improve the Signal to Noise Ratio (SNR). The step-by-step processing during the online phase is described as follows.

#### Data preparation

The noisy recorded post-dive DU data were recently digitized. The presence of noise in the background of the speech signal makes detection and recognition difficult. Power spectral density of non-stationary noise was used and implemented in MATLAB to enhance the noisy speech [40].

The noise estimation is updated by averaging the noisy speech power spectrum using time and frequency dependent smoothing factors. Signal presence is controlled by computing the ratio of the noisy speech power spectrum to its local minimum, which is updated continuously by averaging past values of the noisy speech power spectrum. The enhanced audio is then split into one-minute audio chunks so that it can be fed through the free version of Google speech recognizer for offline processing without being uploaded to the cloud [26, 27].

#### Use of Automatic Speech Recognition (ASR) for real-time transcribing

Traditional speech recognition systems have three major components [41]: the acoustic model, the pronunciation model, and the language model. These components are trained separately but are then merged into one general search graph. The acoustic model recognizes the phonemes that are most likely to be present in raw audio data. It takes a waveform, chunks it into small time segments, implements a frequency analysis, and outputs a probability distribution over all the triphone-states for that input. More recently, deep learning has also enabled the use of end-to-end training of speech recognition systems. Such models, including the Google speech recognizer, substitute the traditional components of an ASR system with a single, end-to-end trained, all neural model that estimates the character sequences directly [42]. After processing each audio segment, the exact timestamps of the Speech components are extracted for each audio chunk to collect the exact location of the human speech. This information is then used to separate the DU (Non-speech component) (useful data) from the Speech component based on the timestamps. This step depends highly on the performance of the noise reduction in the previous step. Higher SNR results in better detection and recognition of the human voice [43].

#### Speech/Non-Speech separation and verification

Once the audio recognition is completed, the algorithm asks the end-user for final verification of separated audio files before saving into the memory. This is a critical step, since the algorithm may not be reliable in all the detections especially when dealing with old data, and in this way the end-user must corroborate the recognition and separation tasks. To help the end-user decide and save the correct files, the spectrograms of separated audio files (Speech and Non-speech components) is shown on the GUI platform as a piece of complementary information. A sample result of the separation step is provided in the next section.

### B. GUI structure

**Fig 4:**
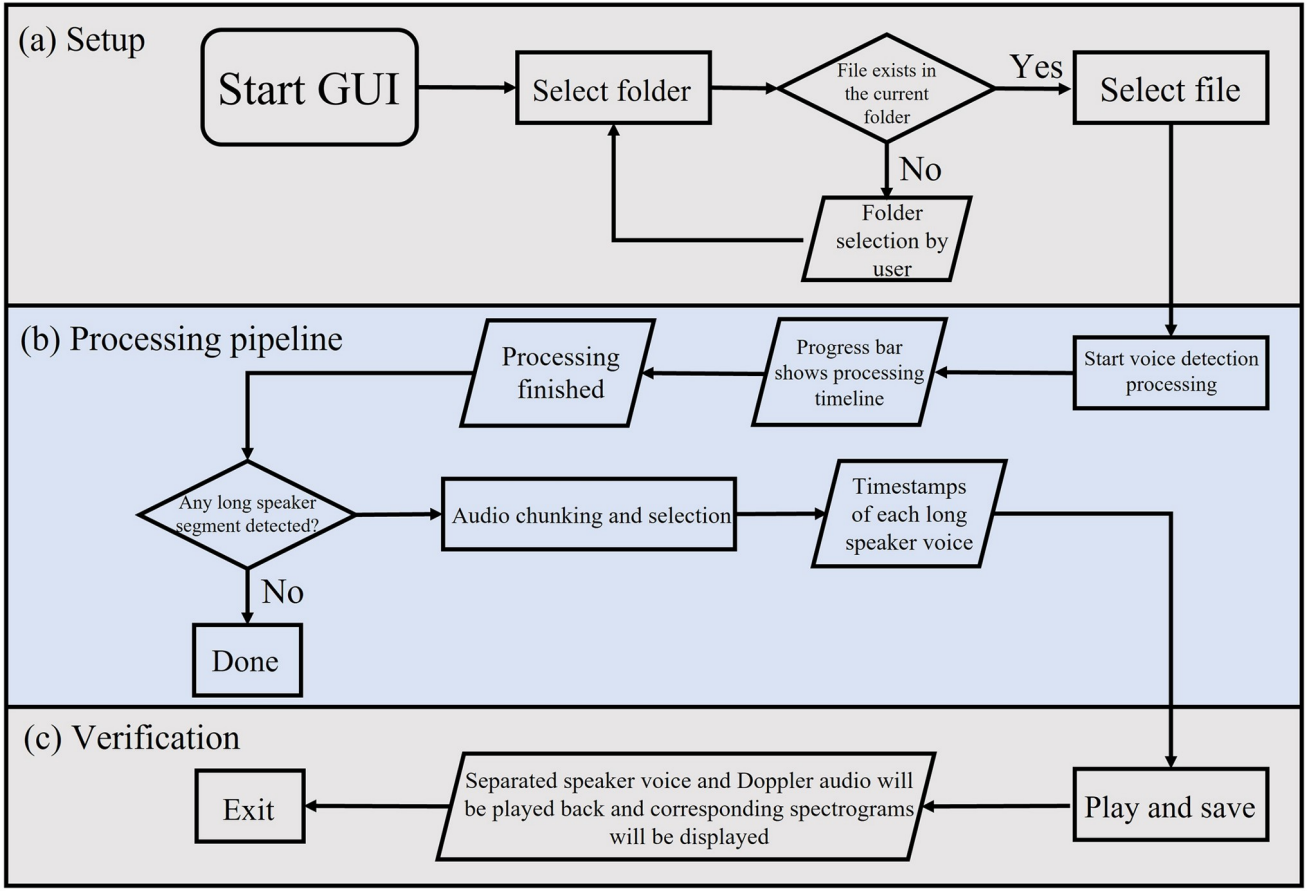
Schematic showing the processing pipeline of the GUI. The main steps are as follows: (a) Setup: Select a file to process from the end-user’s local directory, (b) Processing pipeline: Real-time transcription of the selected audio to obtain timestamps of the speaker voice in long segments, and (c) Verification: The speech component is extracted based on the detected timestamps in the previous step, spectrograms are displayed to help the user decide whether to save the separated components.

A GUI was established and designed using the license-free Tkinter package of Python [44]. Tkinter, or Tk interface, is a Python package that provides an interface to Tk GUI toolkit, and works with common platforms, e.g., MS Windows, Linux, and Mac OS. Event handling, widgets, and geometry management are three major components of Tkinter package. Visual elements are rendered using local operating system elements, so applications built with Tkinter look like they belong on the platform where they are run. We have integrated the Google speech recognition into our GUI and one possible platform to take advantage of it is based on Python programming language, rendering this choice practical for the purpose of this program. Finally, another advantage of using this programming language for building the GUI is that the final program can be packaged into an executable file (.exe) that can then be executed in any Windows-based machine without needing to install Python. This is an attractive feature for our application where this program may be used by users with little or no coding experience. A schematic representation of the developed GUI is shown in Fig 3. The functionality of each button for the developed GUI is provided in the following subsections in detail. The backbone processing of the GUI is shown in Fig 4. It consists of three major components. In the setup phase, the user selects a file to process. The processing pipeline then consists of starting and completing the voice detection process then finding the timestamps associated with the start and end of each detected speech segment if any are detected. Finally, the verification step displays the detected components visually to the user for his/her input with regard to saving.

#### Select file button

**Fig 5:**
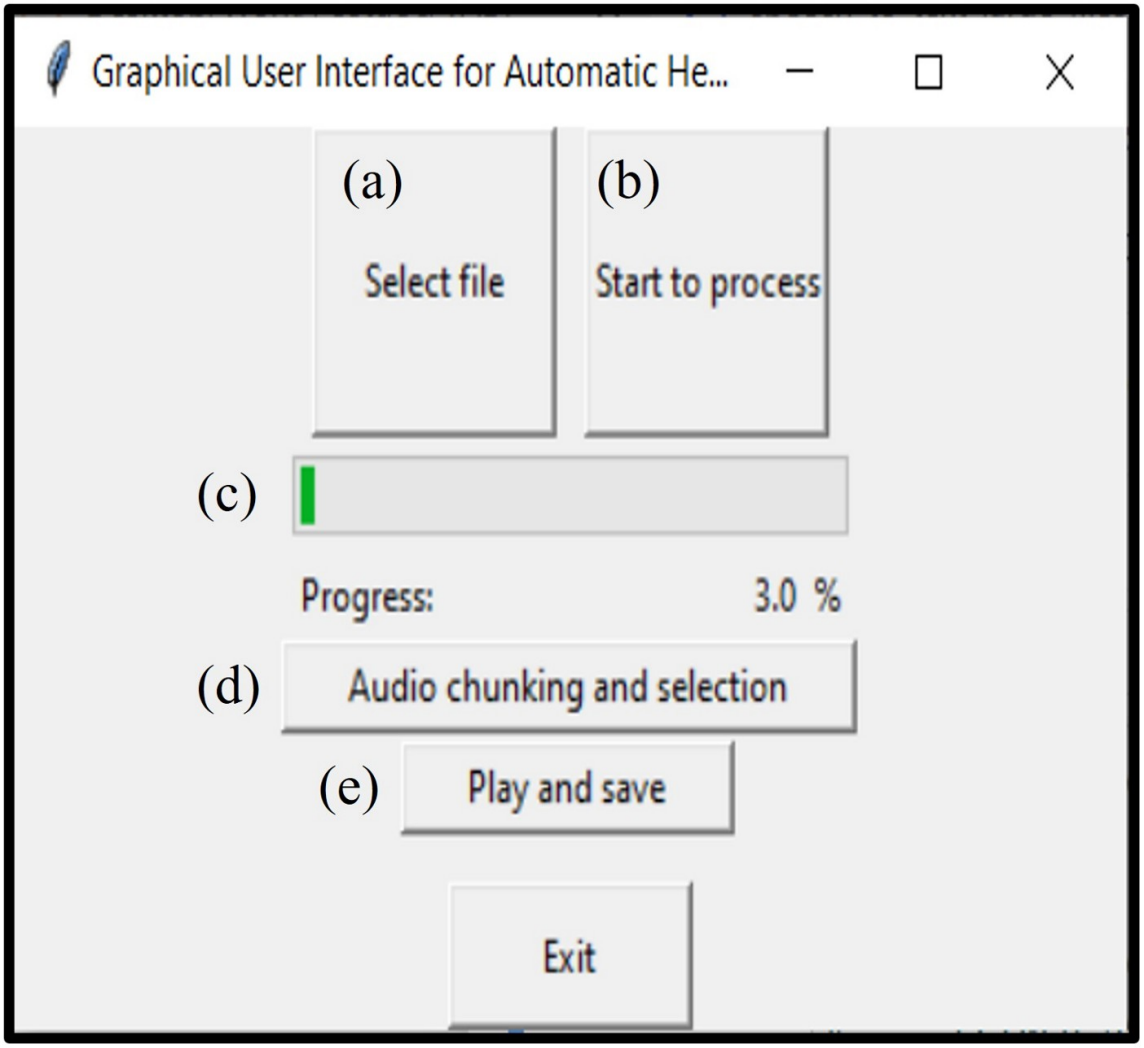
Developed GUI.

**Fig 6:**
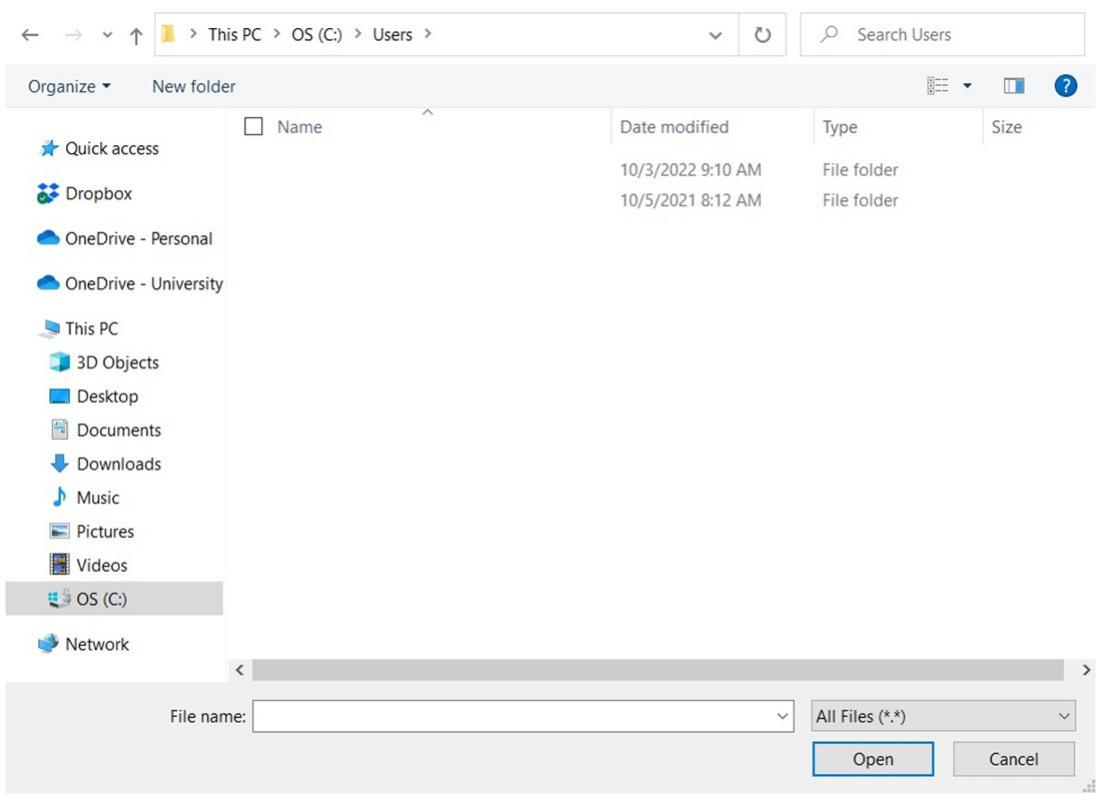
Generic path and audio file selection in GUI.

The first step in the pipeline (shown as part (a) in Fig 5) is to select the input file. By clicking on the Select file button, the generic path in MS Windows will be opened (see Fig 6). At this point, the end-user must select the denoised file for further processing. If the GUI runs in the Command Window as the backbone, then after audio file selection some features of the selected audio file will be printed out, including the samples rate, length of the signal in terms of number of samples along with number of detected one-minute audio chunk. It is worth noting that for the best performance of detection and separation using Google speech recognizer, all the denoised audio files were down sampled to 16 kHz sample rate. Thus, the number of detected one-minute audio chunk mentioned above is simply calculated as the total number of samples divided by sample rate.

#### Start to process button

This button (shown as part (b) in Fig 5) applies the Google speech recognizer to the one-minute audio segments on the fly. The audio segments come from the previous step, during the file selection process. If the GUI runs in the Command Window as the backbone, the detected transcript together with its timestamp will be printed out for each iteration. In case the algorithm cannot find any presence of human voice, it simply prints an empty matrix at the output. The detected transcripts together with the timestamps (starting and ending point of Speech component) are then saved into two different lists during the processing for further analysis. The progress bar shown in Fig 5(c) gets updated once the detection and recognition of an audio segment finishes. When the last audio segment gets processed, the program notifies the end-user to move forward with the next step.

#### Audio chunking and selection button

By clicking on the Audio chunking and selection button, these files get pruned from all detected human voice activity for final verification in the next step.

#### Play and save button

**Fig 7:**
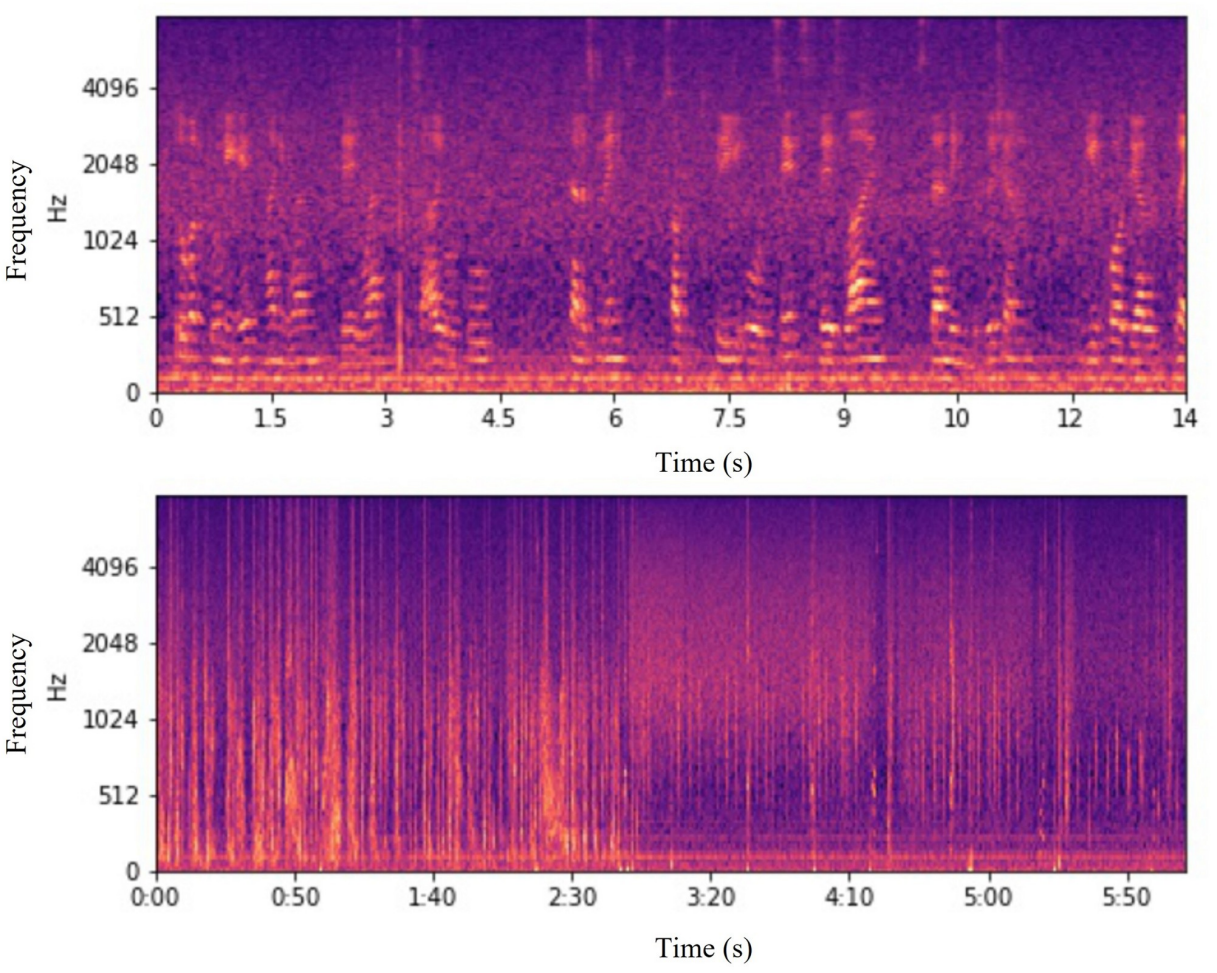
Sample spectrograms of the separated Speech and Non-speech components.

**Fig 8:**
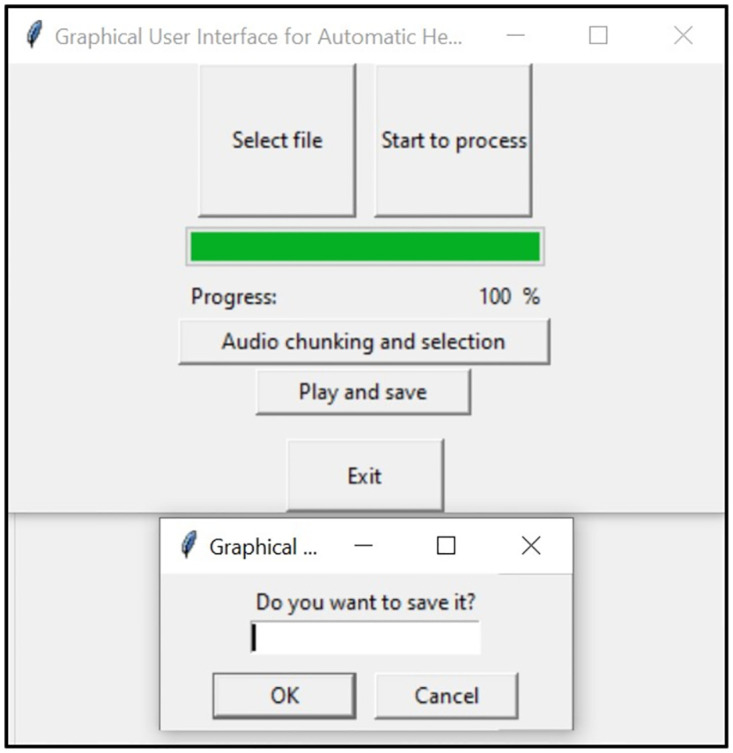
User query box to confirm the separation process and save the resulting separated audio files.

In this step, the separated Speech and Non-speech components are displayed to the end-user. Spectrograms are shown, an example of which can be seen in Fig 7, and the user can also listen to the separated audio if desired. At this stage, the end-user must confirm the effectiveness of the recognition and separation tasks by typing “yes” or “no” in the opened text box, as shown in Fig 8. After confirmation, the separated Speech and Non-speech components get saved into two different folders.

### C. Dataset used and evaluation metrics

The dataset comprised analogue recordings of human precordial and subclavian audio Doppler signals that were made as part of a study conducted by the United States Navy Experimental Diving Unit in the 1980s. The analogue tapes were then converted to a digital format using an analogue to digital converter (Behringer UCA202, Willich, Germany) connected to a PC, where they were saved as digital files in the .flac format. The duration of each audio file is summarized in Table 1. The last column in this table (denoted as no. long segments) accounts for the number of Speech components in each recording. Long segments were defined as Speech of at least three-second duration, where the examiner provides information related to the recording that follows. This is typically done between subjects being recorded, or for the same subject recorded at different times post-dive. In diving research, recordings are often performed both at rest and after movement (e.g. leg flexions). In those cases, the experimenter will briefly speak the words “rest” and “flex”. Here we concentrate on separation between long segments rather than picking up the start and end of rest and flex recordings done consecutively.

**Table 1:** Dataset description.

File ID	Length (minutes)	No. Long Segments
Su204-D1/2-sdA	40	7
Su302-D1/2-sdA	58	19
Su302-D1/2-sdB	40	7
Su302-D3/4-sdA	60	11
Su302-D3/4-sdB	36	6
Su302-D5/6-sdA	49	9
Su302-D5/6-sdB	44	11
Su306-D1/2-sdA	57	9
Su306-D1/2-sdB	30	7
Su306-D3/4-sdA	57	11
Su306-D3/4-sdB	16	3
Su306-D5/6-sdA	52	11
Su306-D5/6-sdB	37	6
**Total**	**576**	**117**

**Fig 9:**
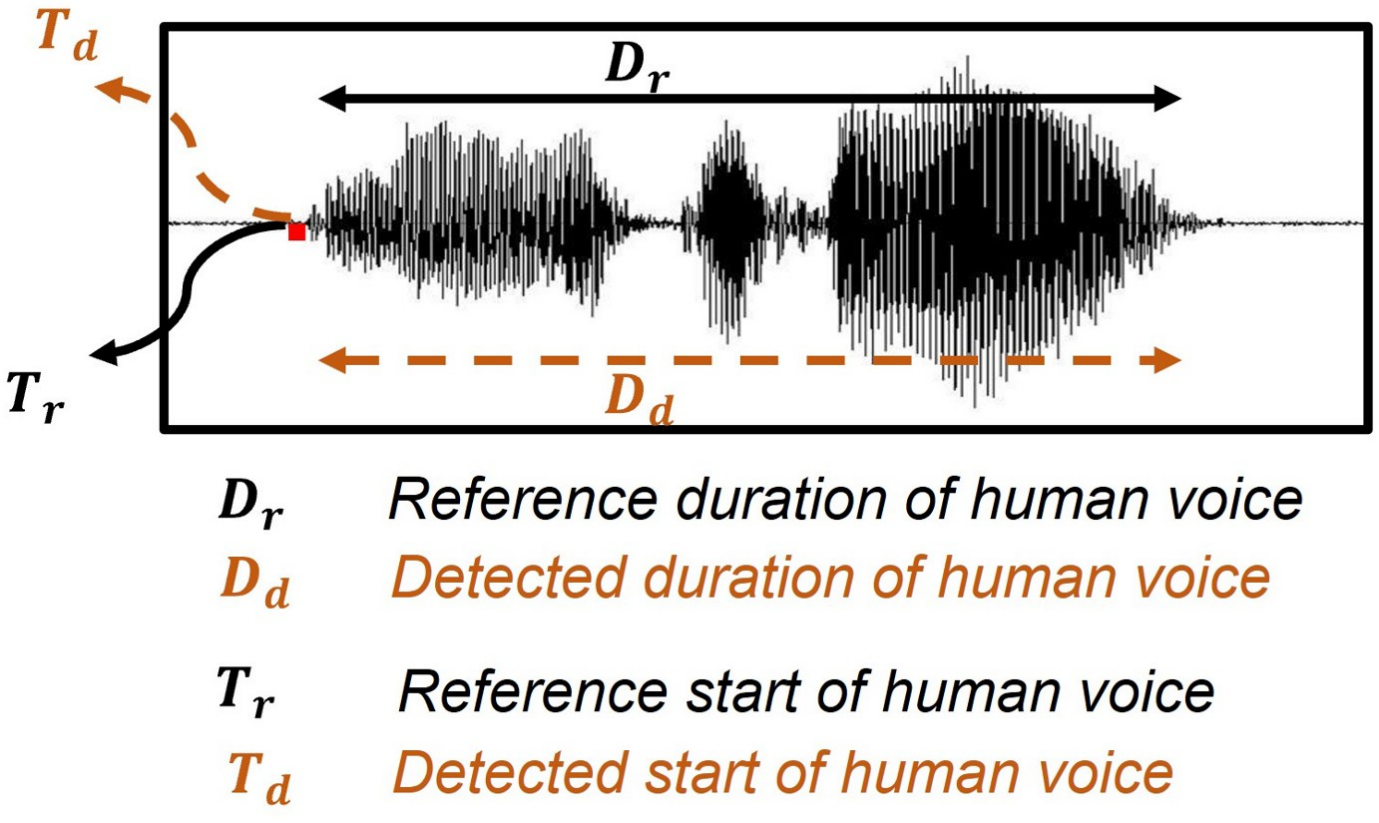
Metrics of algorithm performance assessment.

The start and end points of the Speech component were annotated manually as the reference for the entire dataset to test the effectiveness of the developed algorithm. Two complementary metrics were used for evaluation, as defined in Fig 9: (1) M1: Ratio of total duration of detected Speech component to total duration of reference Speech component, and (2) M2: The absolute difference start of timestamp between the detected Speech component segment and the corresponding reference audio segment. The percentage of Speech component duration correctly identified by the algorithm M1 is defined as:

$$\begin{array}{c} M_{1}=\frac{D_{d}}{D_{r}}\times 100 \end{array}$$ (1)

where D_r_ and D_d_ are the reference duration of Speech component and detected duration Speech component, respectively. Furthermore, the error in start time detection of each voice segment, M_2_, in seconds, is calculated as:

$$\begin{array}{c} M_{2}=\left|T_{d}-T_{r}\right| \end{array}$$ (2)

in which T_r_ and T_d_ represent the reference start of Speech component and detected start of Speech component, respectively.

## Results and discussion

**Fig 10:**
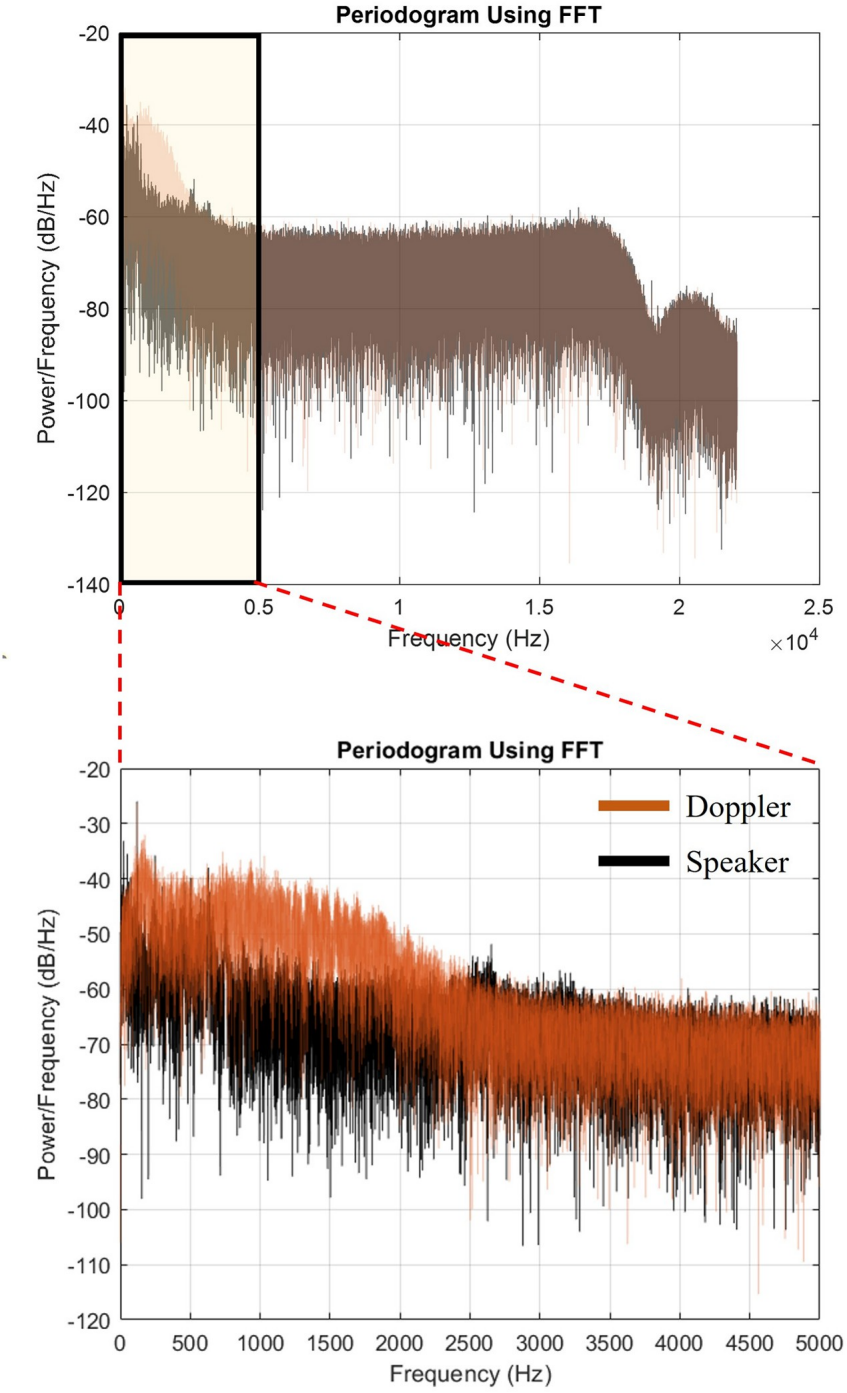
Frequency analysis of speech component and non-speech (Doppler audio) component in a representative recording, showing significant superposition of the frequency content.

The estimation of the power spectral density of the speech and non-speech components in each recording were analyzed, which converts the signal from time domain into the frequency domain. This method was used for the estimation of the power signal at different frequencies in this study. One representative example of the results is shown in Fig 10. The power spectral density (periodogram) of the human voice component and the Doppler audio component are shown in the top row, and a zoomed-in portion comprising the overlaid frequency components between 0 to 5 kHz (where most of the energy exists) is shown in the bottom row. They are shown significantly superimposed, so that more advanced signal processing and learning-based algorithms are required to effectively separate them.

The performance results for different audio files are reported in Table 2. On average, the algorithm was able to detect 79.1% of Speech component throughout the entire dataset. The individual performance per audio file is provided in the 2nd column of Table 2. The average error in detected duration was found to be 3.94 ±2.24 s, with individual audio file results reported in the 3rd column of Table 2. The average error in the start timestamp detection of the Speech component was 2.84 ±1.65 s, and individual audio file results are presented in the 4th column of Table 2.

**Table 2:** Performance results of the developed algorithm.

File ID	Duration detection accuracy–M1 (%)	Duration detection absolute difference error (s)	Start of timestamp absolute error–M2 (s)
Su204-D1/2-sdA	92.0	1.94 ±1.11	0.67 ±0.67
Su302-D1/2-sdA	73.6	5.24 ±5.20	2.59 ±1.68
Su302-D1/2-sdB	82.8	3.21 ±3.58	1.17 ±0.91
Su302-D3/4-sdA	79.8	3.13 ±1.74	1.61 ±0.94
Su302-D3/4-sdB	77.2	4.23 ±2.23	2.1 ±1.45
Su302-D5/6-sdA	77.9	4.16 ±1.67	3.31 ±1.44
Su302-D5/6-sdB	71.6	5.12 ±2.84	2.8 ±2.33
Su306-D1/2-sdA	76.7	4.46 ±1.12	3.23 ±1.95
Su306-D1/2-sdB	75.2	4.55 ±2.1	4.44 ±2.5
Su306-D3/4-sdA	78.9	4.24 ±2.44	3.36 ±2.11
Su306-D3/4-sdB	87.4	2.5 ±1.47	3.23 ±2.32
Su306-D5/6-sdA	76.6	4.61 ±1.88	3.81 ±1.74
Su306-D5/6-sdB	78.6	3.93 ±1.76	4.35 ±1.47
**Total**	**79.1**	**3.94 ±2.24**	**2.84 ±1.65**

**Fig 11:**
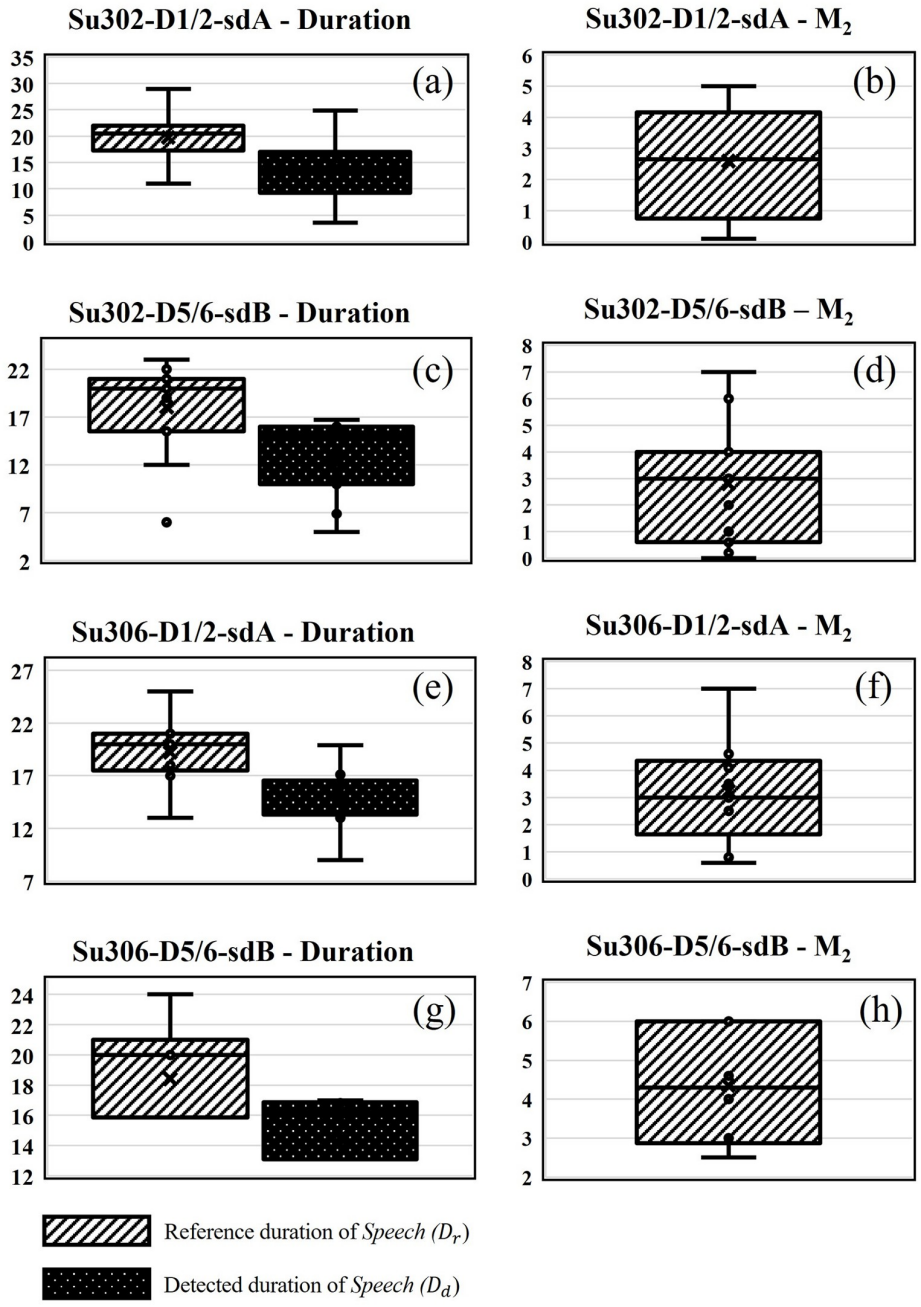
Performance of the developed model in terms of duration error and start of timestamp error for better visualization in representative samples.

To show the effectiveness of the developed algorithm visually and make the interpretation of the results easier, four sample audio files were randomly selected, and the bar chart of system performance is plotted in Fig 11. The first column of Fig 11 shows the duration error for the selected audio files and the second column corresponds to the error of the start of timestamp. The current system achieves a good performance at 79.1% of voice segments recognized. However, one limitation of the Google Speech recognizer is that voice segments of insufficient quality for speech recognition may be missed. This was addressed in our work through the initial denoising step. In the future, we could investigate the performance of voice activity detection (VAD) algorithms as an alternative strategy [45], since those do not focus on word recognition but rather the differences in acoustic features.

While not a perfect surrogate marker for DCS [39], VGE remain to date the most widely used decompression stress marker in physiological studies. Used appropriately, they are an important component of decompression physiology and pathophysiology research. Efforts such as the one detailed in this work aim to leverage the abundance of historic data collected in the field. In parallel, additional initiatives are needed to further discussions within the diving research community to develop testable hypotheses on large databases and other candidate biomarkers.

## Conclusion

Leveraging previously collected DU data is especially important in diving research due to the difficulty of repeating large-scale military hyperbaric exposures that were conducted in the 70–90s in austere environments. Historically, these were often collected on cassettes as one-channel audio with superimposed human speech describing the experiment, making digitization and separation of these audio files a lengthy, manual task. Since the processing of this data relies heavily on the effective separation of the human voice from the ultrasound audio, we have developed a novel graphical user interface (GUI) to aid in these recognition and separation tasks. We used the Google speech recognizer within our developed GUI to extract the timestamps of Speech component and perform separation. Speech separation technology has not previously been used in post-dive Doppler ultrasound recordings. Here we show promising preliminary performance for its capacity to help separate long back to back recordings that could help accelerate the reuse of large amounts of unique previously-collected data. The developed algorithm tested on our private domain dataset shows that the recognition and separations tasks are performed with good accuracy. This may allow a human operator to save time in reviewing historic cassettes, where the approximate times of Speech/Non-speech transition are presented, and they can selectively listen to those to expedite manual separation of the segments of interest.

## Supporting information

S1 File Raw data supplementary file. (PDF)
