# Supplementary material for: Development of a graphical user interface for automatic separation of human voice from Doppler ultrasound audio in diving experiments
Source: PLoS One. 2023 Aug 10;18(8):e0283953. doi: 10.1371/journal.pone.0283953 (PMC10414643; doi:10.1371/journal.pone.0283953)
Supplement: S1 File — (PDF) [file pone.0283953.s001.pdf]

Raw Data Supplementary File

Title: Raw Data for Su204-D1/2-sdA

Duration Comparison

Reference	Detected	Error
18.6	14.5	4.1
23	21	2
23	21.5	1.5
22	19.4	2.6
25	24.1	0.9
24	23	1
39	37.5	1.5

Start of timepoint comparison

Start - Reference	Start - Detected	Difference
10	10.2	0.2
21	22	1
30	29.9	0.1
35	35.3	0.3
9	8.2	0.8
28	28.3	0.3
28	26	2

Title: Raw Data for Su302-D1/2-sdA

Duration Comparison

Reference	Detected	Error
15.29	14.9	0.39
22	22	0
29	24.9	4.1
11	3.6	7.4
21	13.4	7.6
23	23	0
22	12	10
13	13.6	-0.6
20	14.4	5.6
21	14.8	6.2
20	13.6	6.4
18	4.6	13.4
21	13.4	7.6
17	17.7	-0.7
18	8.3	9.7
25	7	18
21	21	0
20	18.5	1.5
20	17.1	2.9

Start of timepoint comparison

Start - Reference	Start - Detected	Difference
1.11	0.8	0.31
52	51.9	0.1
23	24.4	1.4
43.5	48	4.5
21	25.3	4.3
19	17	2
34	39	5
3	2.4	0.6
50	53	3
48	52	4
10	14.3	4.3
18	22.1	4.1
14	18	4
21	20.2	0.8
23	26.5	3.5
7	9.3	2.3
24	23.6	0.4
2	0	2

Title: Raw Data for Su302-D1/2-sdB

Duration Comparison

Reference	Detected	Error
14	14.9	-0.9
25	18	7
23	16	7
22.6	16	6.6
16	13.4	2.6
23	23	0
7	6.8	0.2

Start of timepoint comparison

Start - Reference	Start - Detected	Difference
9	9.2	0.2
0	0.1	0.1
55	54.1	0.9
37.4	36	1.4
53	51	2
20	17.4	2.6
57	58	1

Title: Raw Data for Su302-D3/4-sdA

Duration Comparison

Reference	Detected	Error
13	9	4
22.1	22	0.1
24	15	2
20	17	3
24	21	3
17	15	2
22	19	3
22	16	6
26	20	6
11	7.6	3.4
4	2	2

Start of timepoint comparison

Start - Reference	Start - Detected	Difference
5	4.2	0.8
24.5	23	1.5
8	6	2
32	28	4
7	6.4	0.6
13	11	2
27	26	1
26	24.3	1.7
9	7.9	1.1
20	19	1
44	42	2

Title: Raw Data for Su302-D3/4-sdB

Duration Comparison

Reference	Detected	Error
16.6	11	5.6
22	20	2
20	14	6
22	15	7
22	19	3
9	7.2	1.8

Start of timepoint comparison

Start - Reference	Start - Detected	Difference
4	3.8	0.2
2	3.4	1.4
34	30	4
37	36	1
38	35	3
29	26	3

Title: Raw Data for Su302-D5/6-sdA

Duration Comparison

Reference	Detected	Error
10.6	6	4.6
26	20	6
20	15.6	4.4
20	17	3
22	19	3
27	20	7
21	18.5	2.5
18	13	5
5	3	2

Start of timepoint comparison

Start - Reference	Start - Detected	Difference
3.8	2	1.8
50	46	4
37	32	5
46	42	4
22	20	2
1	4	3
50	46	4
26	25	1
48	43	5

Title: Raw Data for Su302-D5/6-sdB

Duration Comparison

Reference	Detected	Error
15.5	15	0.5
21	16	5
20	12.6	7.4
23	16	7
20	10	10
19	16	3
19	11.9	7.1
22	16	6
21	16.7	4.3
12	6.9	5.1
6	5	1

Start of timepoint comparison

Start - Reference	Start - Detected	Difference
3.8	3.6	0.2
34	40	6
12	12	0
12	13	1
41	48	7
14	13.4	0.6
46	43	3
19	15	4
42	40	2
15	11	4
16	13	3

Title: Raw Data for Su306-D1/2-sdA

Duration Comparison

Reference	Detected	Error
13	9	4
20	14.4	5.6
21	14.8	6.2
18	13.6	4.4
21	16	5
17	13	4
18	15	3
25	19.9	5.1
20	17.1	2.9

Start of timepoint comparison

Start - Reference	Start - Detected	Difference
3	3.6	0.6
47	50	3
46	50.1	4.1
10	13	3
18	22.6	4.6
14	16.5	2.5
21	20.2	0.8
23	26.5	3.5
40	47	7

Title: Raw Data for Su306-D1/2-sdB

Duration Comparison

Reference	Detected	Error
14.5	13.6	0.9
23	16	7
22	15	7
20	16	4
22	18	4
21	16	5
6	2	4

Start of timepoint comparison

Start - Reference	Start - Detected	Difference
0.5	3.6	3.1
27	33	6
22	20	2
29	25	4
24	22	2
55	50	5
56	47	9

Title: Raw Data for Su306-D3/4-sdA

Duration Comparison

Reference	Detected	Error
12.5	8	4.5
21	15	6
23	16	7
20	16.3	3.7
19	12.6	6.4
33	26	7
19	18	1
23	17	6
22	21	1
22	19	3
6	5	1

Start of timepoint comparison

Start - Reference	Start - Detected	Difference
4	3	1
52	58	6
46	48	2
1	5	4
14	13	1
11	10	1
30	37	7
18	16	2
14	10	4
52	48	4
55	50	5

Title: Raw Data for Su306-D3/4-sdB

Duration Comparison

Reference	Detected	Error
15.2	14	1.2
22.2	20	2.2
22.1	18	4.1

Start of timepoint comparison

Start - Reference	Start - Detected	Difference
3.9	6	2.1
43.3	45	1.7
59.1	65	5.9

Title: Raw Data for Su306-D5/6-sdA

Duration Comparison

Reference	Detected	Error
11.1	8	3.1
19	16	3
19	14.5	4.5
21	15	6
22	19	3
21.7	20	1.7
24.1	16.5	7.6
21.4	15.7	5.7
15	11	4
20.8	13.6	7.2
21.8	16.9	4.9

Start of timepoint comparison

Start - Reference	Start - Detected	Difference
3.3	6	2.7
55.1	59	3.9
14	19	5
43	46	3
18	25	7
51	53	2
40	45	5
41.5	43	1.5
9	14	5
53	58	5
24.2	26	1.8

Title: Raw Data for Su306-D5/6-sdB

Duration Comparison

Reference	Detected	Error
24	16.8	7.2
20	17	3
20	15.8	4.2
18.5	16.3	2.2
20	16	4
8	5	3

Start of timepoint comparison

Start - Reference	Start - Detected	Differenc e
21	25.6	4.6
7	9.5	2.5
23	26	3
41	47	6
18	24	6
17	21	4
